# Effects of antibacterial peptides on rumen fermentation function and rumen microorganisms in goats

**DOI:** 10.1371/journal.pone.0221815

**Published:** 2019-08-30

**Authors:** Zhihua Ren, Renjie Yao, Qi Liu, Youtian Deng, Liuhong Shen, Huidan Deng, Zhicai Zuo, Ya Wang, Junliang Deng, Hengmin Cui, Yanchun Hu, Xiaoping Ma, Jing Fang

**Affiliations:** The Key Laboratory of Animal Disease and Human Health of Sichuan Province, Key Laboratory of Environmental Hazard and Animal Disease of Sichuan Province, College of Veterinary Medicine, Sichuang Agricultural University, Chengdu Sichuang, China; Universite Paris-Sud, FRANCE

## Abstract

Although many studies have confirmed that antimicrobial peptides (AMPs: PBD-mI and LUC-n) can be used as feed additives, there are few reports of their use in ruminants. The present study aimed to investigate the impact of AMPs on ameliorating rumen fermentation function and rumen microorganisms in goats. Eighteen 4-month-old Chuanzhong black goats were used in a 60-day experiment (6 goats per group). Group I was used as the control and was fed a basal diet, the group II were fed the basal diet supplemented with 2 g of AMPs [per goat/day] and group III were fed the basal diet supplemented 3 g of AMPs [per goat/day], respectively. Rumen fluid samples were collected at 0, 20 and 60 days. Bacterial 16S rRNA genes and ciliate protozoal 18S rRNA genes were amplified by PCR from DNA extracted from rumen samples. The amplicons were sequenced by Illumina MiSeq. Rumen fermentation parameters and digestive enzyme activities were also examined. Our results showed that dietary supplementation with AMPs increased the levels of the bacterial genera *Fibrobacter*, *Anaerovibrio* and *Succiniclasticum* and also increased the ciliates genus *Ophryoscolex*, but reduced the levels of the bacterial genera *Selenomonas*, *Succinivibrio* and *Treponema*, and the ciliate genera *Polyplastron*, *Entodinium*, *Enoploplastron* and *Isotricha*. Supplementation with AMPs increased the activities of xylanase, pectinase and lipase in the rumen, and also increased the concentrations of acetic acid, propionic acid and total volatile fatty acids. These changes were associated with improved growth performance in the goats. The results revealed that the goats fed AMPs showed improved rumen microbiota structures, altered ruminal fermentation, and improved efficiency regarding the utilization of feed; thereby indicating that AMPs can improve growth performance. AMPs are therefore suitable as feed additives in juvenile goats.

## 1 Introduction

The microbiota colonizing the rumen is an essential component of ruminant gastrointestinal tract(GIT)[1]. The microbial community in the rumen consists of bacteria (10^10^–10^11^ cells/mL), methanogenic archaea (10^7^–10^9^ cells/mL), ciliate protozoa (10^4^–10^6^ cells/mL), anaerobic fungi (10^3^–10^6^ cells/mL) and bacteriophages (10^9^–10^10^ particles/mL)[2]. A major function of the rumen microbiome is the fermentation of plant materials ingested by ruminant animals[3–5]. Rumen modulation is one of the most important methods for improving feed efficiency, ruminant health and performance in ruminant livestock production. Several antibiotic compounds, such as monensin, hainanmycin and virginiamycin, have been used to improve ruminal fermentation and the efficiency of nutrient utilization[6–8]. However, the overuse of antibiotics has raised concerns about product safety and environmental health, and the use of antibiotics as additives in animal feed is banned in the European Union (European Union, 2003).

Antimicrobial peptides (AMPs) are widespread in all living cells. They are endowed with antimicrobial[9], antifungal[10], antiviral[11], anti-parasitic[12] and antitumor activities[13]. Furthermore, immunoregulatory and antioxidant activities induced by AMPs have been shown to be mediated by the cationic charge, amphipathicity, amino acid composition and structure of these peptides[14]. AMPs act against target organisms either by membrane depolarization, micelle formation or the diffusion of AMPs onto intracellular targets[15–18]. Until now, few studies have reported on the use of AMPs as alternatives to feed antibiotics and growth promoters in ruminant nutrition. Nonetheless, AMPs have been associated with improved performance, nutrient retention and intestinal morphology, and to reduce the incidence of diarrhea in weanling piglets[19–22]. Peng *et al*.[23] demonstrated that dietary supplementation with crude recombinant porcine β-defensin 2 (rpBD2) has beneficial effects on the growth and intestinal morphology of weaned piglets, reducing the incidence of post-weaning diarrhea and the number of potential pathogens in the caecum. Therefore, AMPs are likely to serve as potential alternatives to antibiotics in livestock production[19]. Previous studies in our laboratory showed that adding AMPs (recombinant swine defensin and a fly antibacterial peptide mixed at a 1:1 ratio) to feed could improve the growth performance and immunity of weaned piglets[14,24]. Based on our previous findings and the reported bactericidal effects of AMPs, we hypothesized that dietary AMP supplementation could affect the rumen microbiota, and therefore ruminal fermentation. In the present study, we investigated the effects of AMPs on rumen fermentative function and rumen microbial community structure in Chuanzhong black goats.

## 2 Materials and methods

### 2.1 Materials

The antimicrobial peptides (AMPs) used in the present study were provided by Rota BioEngineering Co., Ltd. (Sichuan, China). The AMPs were composed of recombinant swine defensin PBD-mI (DHYICAKKGGTCNFSPCPLFNRIEGTCYSGKAKCCIR), the net charge was caculated using protein calculator v3.4 (estimated charge at pH 7.00 = 4.0), and a molecular mass of about 5.4 kDa was obtained through a codon-optimized protein corresponding to mature defensin cDNA that was expressed and purified in *Pichia pastoris* yeast[25], and a fly antibacterial peptide LUC-n (ATCDLLSGTGVKHSACAAHCLLRGNRGGYCNGRAICVCRN), the net charge was caculated using protein caculator v3.4(estimated charge at pH 7.00 = 4.2) and a molecular mass of approximately 21.18 kDa was obtained through complementary DNA (cDNA) libraries constructed from micro-dissected salivary glands in whole maggots, that underwent transposon-assisted signal trapping, a technique selected for the identifi-cation of secreted proteins[26], at a blending ratio of 1:1[14]. The purity of both components was estimated to be over 93%, which was purified by RP-HPLC and analysed by SDS-PAGE(Provided by Rota BioEngineering Co.,Ltd.). Each preparation was stored at dry, ventilated and lightproof place.

### 2.2 Animal handling

The experimental procedures performed on the goats and the care of the animals were approved by the Guide for Sichuan Agricultural University Animal Care and Use Committee, Sichuan Agricultural University, Sichuan, China, under permit no. DKY-B20100805, The young goats used in this study were healthy.

The AMPs used in this study were added to basal diets, using a portion of the basal diet as a carrier. Due to the previous study(+1g/kg of AMPs) by our research group[14], we intend to detect the effects of larger amount of the AMPs on rumen. The AMPs were mixed with carrier (basal diet) such that the addition of 2 and 3 g/kg of AMP with carrier equated to 30 and 45 mg/kg of dietary AMPs, respectively.

Eighteen uncastrated 4-month-old Chuanzhong black goats (*Capra hircus*; average weight 15.52±0.35 kg) were acclimated for 7 days before the experiment and received a basal diet(NRC,2007) only to ensure that the daily diet was fully consumed. All goats were caged, randomly organized into three groups, and were maintained at 25±2°C with a 10 h light-14 h dark cycle. Group I was the control group; group II received a basal diet + 2 g of AMPs per head per day; and group III received a basal diet + 3 g of AMPs per head per day. The diet included concentrate (300 g per head per day) (Table 1) and forage (fresh grass, *Zoysia japonica*, 300 g per head per day), after finishing the concentrate. The forage that was refused was collected and weighed every second morning (at 8:00) to record the intake of forage per group per day. Animals were housed with free access to water, and fed individually twice daily (at 09:00 and 18:00); the animals maintained their normal herd behavior. Of the goats fed diets containing AMPs, all consumed the complete daily concentrate diet under our daily supervision.

**Table 1:** Composition and nutrient levels of the concentrate (DM basis).

Ingredients	Content(%)	Nutrient levels	Content(%)
Corn grain	51	Digestive Energy / (MJ / kg)	13.38
Wheat bran	23	Dry Matter	84.27
Rapeseed meal	10	Crude Protein	16.59
Rapeseed cake	10	Crude Fiber	4.14
Fish meal	3	Neutral Detergent Fiber	13.66
NaCl	1	Acid Detergent Fiber	6.94
Premix^1)^	2		
Total	100		

^1)^Premix provides the following per kg of the diet: Fe (as ferrous sulfate) 30 mg, Cu (as copper sulfate) 10 mg, Zn (as zinc sulfate) 50 mg, Mn (as manganese sulfate) 60 mg, VitaminA 2 937 IU, VitaminD 343 IU, VintaminE 30 IU.

### 2.3 Sampling and DNA extraction

Rumen fluid samples were collected using a stomach tube on days 0, 20 and 60, prior to morning feeding; the first part of the rumen fluid was discarded to prevent interference from saliva. Three goats were selected from each treatment group for sampling (50 mL/goat). The rumen pH was measured immediately after collection using a portable pH meter[27] (PHB-4, Shanghai Leica Scientific Instrument Co., Ltd., Shanghai, China). Solid feed particles were removed from the rumen fluid by filtration through four layers of cheesecloth. Then, 5 mL of the ruminal fluid was mixed with 1 mL of 25% (w/v) meta-phosphoric acid and the mixture was stored at ˗80°C for later analysis, including the analysis of volatile fatty acids (VFAs). Microbial genomic DNA was extracted from the rumen samples using a stool DNA kit (OMEGA Bio-Tek, Norcross, GA, USA), in accordance with the manufacturer’s instructions[5].

### 2.4 Ruminal fermentation function analysis

The frozen samples were thawed at 4°C and centrifuged at 3000×g for 10 min. The supernatant was mixed with the same volume of 20 mM 4-methyl N-valeric acid as an internal standard in preparation for total-VFA (T-VFA) analysis and chromatography according to Luo *et al*.[28]. The concentration of NH_3_-N was analyzed using visible-light spectrophotometry (Scientific BioMate 3s, Thermo). NH_4_Cl standards were prepared according to Broderick and Kang[29]. The microbial protein (MCP) in the rumen was analyzed by means of TCA protein precipitation[30]. The activities of carboxymethyl cellulase (CMCase), xylanase, pectinase and β-glucosidase were measured using the corresponding commercially available ELISA kits (R&D Systems). Protease activity was measured as follows: a reaction mixture containing 1 mL of casein and 4 mL of protease enzyme was incubated for 4 h at 38°C. Then, the reaction was stopped by the addition of 10% trichloroacetic acid and the sample was centrifuged at 3500×g for 15 min. Next, 1 mL of supernatant was removed and mixed with 5 mL of 0.4 mol/L Na_2_CO_3_ and 1 mL of Folin–Ciocalteu’s phenol solution and incubated on the laboratory bench for 15 min. The hydrolyzed protein was measured using visual-light spectrophotometry at 680 nm. The concentration and activity of lipase and amylase were measured using commercially available reagent kits (NanJing JianCheng Bioengineering Institute, Nanjing, China).

### 2.5 Rumen microbial community analysis

The V4 regions of bacterial *16S rRNA* genes and ciliate protozoal *18S rRNA* genes were amplified. Bacterial sequences were amplified using the primers: 520F 5′-GCACCTAAYTGGGYDTAAAGNG-3′ and 802R 5′-TACNVGGGTATCTAATCC-3′; the ciliate sequences were amplified using the primers V547F 5′-CCAGCASCYGCGGTAATTCC-3′ and V4R 5′-ACTTTCGTTCTTGATYRA-3′. The bacterial amplification mixture consisted of 1 μL (10 μM) of each primer, 1 μL of template DNA, 5 μL of 5×reaction buffer, 5 μL of 5×high GC buffer, 0.5 μL of 10 mM dNTPs, 0.25 μL of Q5 high-fidelity DNA polymerase and 11.25 μL of ddH_2_. The ciliate PCR was carried out in triplicate using 25 μL mixtures containing 1 μL (10 μM) of each primer, 2 μL of template DNA, 5 μL of 5×Q5 reaction buffer, 5 μL of 5×Q5 GC high enhancer, 2 μL of 2.5 mM dNTPs and 0.25 μL (5 U/μL) of Q5 polymerase. Amplification was performed as follows: initial denaturation at 98°C for 5 min; then 27 cycles of denaturation at 98°C for 30 s, annealing at 50°C for 30 s, and elongation at 72°C for 30 s; plus a final 5-min extension step at 72°C. PCR products were excised from 2% agarose gels and purified with a QIAquick gel extraction kit (Qiagen, Venlo, The Netherlands)[5]. The remaining DNA was stored at ˗20°C prior to sequencing. High quality DNA was sent to Shanghai Paisennuo Biological Technology Co. Ltd. (Shanghai, China) for sequencing using an Illumina MiSeqPE250.

### 2.6 Data analysis

Sequences obtained in this study were deposited in the NCBI Sequence Read Archive under BioProject numbers PRJNA398687, PRJNA398591 and PRJNA398697. Sequence reads were processed and analyzed by QIIME pipeline software (version 1.8.0)[31]. Chimeric sequences were removed to generate high quality sequences (UCHIME through the QIIME software). Using the UCLUST sequence alignment tool within the QIIME pipeline software, the high quality sequence was divided and aligned into operational taxonomic units (OTUs) with 97% sequence similarity. The most abundant sequences were compared with template regions in the Greengenes database (Release 13.8, http://greengenes.secondgenome.com/) (bacterial) and the NCBI (http://www.ncbi.nlm.nih.gov) database (ciliate protozoal) to acquire taxonomic information for each OTU and species composition information. Alpha diversity indexes (including the Simpson index and Shannon index) were obtained using QIIME pipeline software. R software was used to analyze microbial population structures. The results of these various analyses are expressed as the means and standard errors of the means (SEM). Statistical comparisons were made by one-way analysis of variance (ANOVA) using a statistical software package (SPSS 19.0, Business Machines Corporation, Armonk, NY, USA). Differences among treatments were regarded as significant at *P* < 0.05.

## 3 Results

### 3.1 Growth performance

The growth performance for all groups of juvenile goats tested is listed in Table 2. Throughout the experimental period, the body weights were higher in the AMP-treated groups than in the control group. The average daily gain (g) was significantly higher (*P <* 0.05) in group II than in group III or the control group. No significant difference in average daily feed intake of forage was found between the AMP-treated groups and the control (*P* > 0.05).

**Table 2:** Changes in the body weight and average daily gain of goats.

Item	Time point(day)/Time range	I	II	III
Weight (kg)	0d	15.50±0.20	15.55±0.45	15.50±0.47
	20d	16.84±0.23^B^	17.95±0.43^A^	17.46±0.56^A^^B^
	60d	18.93±0.28^C^	21.81±0.36 ^A^	19.93±0.54^B^
average daily gain (g/d)	0d-20d	65.63±3.70^C^	120.00±7.00^A^	99.50±4.50^B^
	20d-60d	52.88±2.80^A^	96.51±5.3^B^	61.19±5.10^A^
	0d-60d	57.12±1.3^C^	104.33±2.2 ^A^	73.96±3.4^B^
average daily feed intake of forage (kg/d)	0d-20d	1.18±0.01	1.19±0.02	1.18±0.01
	20d-60d	1.37±0.0	1.36±0.02	1.37±0.02
	0d-60d	1.31±0.01	1.30±0.02	1.31±0.01

^A,B,C^Values with different superscripts in the same row differ significantly (*P<*0.05); I, control group; II and III, groups treated with 2 and 3 g/per head per day of antimicrobial peptides, respectively.

### 3.2 Ruminal fermentation function

#### 3.2.1 Ruminal fermentation parameter

The mean ruminal pH of samples from AMP-treated goats ranged from 6.81 to 6.92, which was within the normal physiological range. No significant difference in ruminal pH between the AMP-treated groups and the control group was observed (*P >* 0.05) (Table 3).

**Table 3:** Changes in the ruminal fermentation parameters in the rumen fluid of goats.

Parameter		I	II	III
pH	0 d	6.89±0.02	6.88±0.03	6.89±0.02
	20 d	6.88±0.04	6.81±0.04	6.82±0.06
	60 d	6.94±0.02	6.90±0.06	6.93±0.05
T-VFA (mmol/L)	0 d	75.42±1.18	75.65±1.48	75.46±0.82
	20 d	69.26±1.32^C^	93.17±0.75 ^A^	88.82±2.04^B^
	60 d	63.97±1.77^C^	82.53±2.58^A^	68.82±2.13^B^
Acetate (mmol/l)	0 d	51.53±1.45	51.63±2.16	51.61±0.91
	20 d	46.18±1.71^B^	64.32±1.84^A^	61.10±2.32^A^
	60 d	41.94±1.5^B^	55.86±2.82 ^A^	45.50±2.35^B^
Propionate (mmol/l)	0 d	15.29±0.36	15.41±0.35	15.27±0.20
	20 d	14.38±0.57^B^	20.14±0.75^A^	19.03±0.99^A^
	60 d	13.24±0.47^C^	17.75±0.45 ^A^	14.87±0.44^B^
Butyrate (mmol/l)	0 d	8.60±0.10	8.61±0.24	8.58±0.16
	20 d	8.70±0.37	8.73±0.38	8.69±0.30
	60 d	8.80±0.40	8.91±0.68	8.90±0.59
Acetate+Butyrate to Propionate ratio	0 d	3.93±0.18	3.91±0.04	3.94±0.09
	20 d	3.82±0.20	3.63±0.20	3.68±0.28
	60 d	3.83±0.22	3.65±0.13	3.66±0.14
MCP (mg/mL)	0 d	1.30±0.06	1.31±0.06	1.31±0.03
	20 d	1.33±0.01	1.40±0.05	1.37±0.08
	60 d	1.34±0.02	1.38±0.06	1.34±0.03
Ammonia (mg/100mL)	0 d	11.20±0.21	11.16±0.22	11.18±0.35
	20 d	9.56±0.31^A^	8.45±0.18^B^	8.89±0.33^B^
	60 d	10.28±0.17^A^	9.41±0.17^B^	9.65±0.37^B^

^A,B,C^Values with different superscripts in the same row differ significantly (*P<*0.05); I, control group; II and III, groups treated with 2 and 3 g/per head per day of antimicrobial peptides, respectively. (The table are detailed in S1 File)

#### 3.2.2 Enzyme activity

Xylanase, pectinase and lipase activities were higher in the AMP-supplemented goats than those in the control group (Table 4; *P* < 0.05). No differences in CMCase or protease activities were observed between AMP-treated goats and the control group (*P* > 0.05). The activities of β-glucosidase and amylase in group II were not significantly different from those in the controls (*P* > 0.05). However, the activities of these enzymes were significantly lower in group III than those in group II or the control (*P* < 0.05).

**Table 4:** Changes in the activity of enzymes in the rumen fluid of goats.

Parameter		I	II	III
CMCase (U/mL)	0 d	74.33±1.59	73.46±1.90	73.46±1.42
	20 d	85.88±2.37	82.19±3.19	81.02±2.87
	60 d	113.41±3.85	110.86±4.39	107.42±3.08
Xylanase (U/mL)	0 d	10.04±0.32	10.01±0.34	10.02±0.38
	20 d	14.57±0.44^C^	25.13±2.08^A^	18.14±1.46^B^
	60 d	21.35±0.61^B^	35.36±1.37^A^	34.57±2.71^A^
Pectinase (U/mL)	0 d	45.51±2.98	45.22±1.68	45.13±2.10
	20 d	37.48±4.52^B^	69.17±4.57^C^	59.76±1.16^A^
	60 d	17.18±2.56^B^	26.91±1.85^A^	26.69±0.60^A^
β-glucosidase (U/ mL)	0 d	72.59±3.22	72.62±3.10	72.51±3.17
	20 d	68.95±4.64^A^^B^	72.03±4.77^A^	62.18±2.47^B^
	60 d	61.69±0.12^A^	63.85±2.43^A^	55.98±3.19^B^
Protease (μg /min^.^mL^-1^)	0 d	3.25±0.74	3.08±0.34	3.11±0.30
	20 d	3.31±0.74	3.13±0.45	3.22±0.31
	60 d	4.48±0.45	4.44±0.57	4.40±0.17
Amylase (U/dL)	0 d	20.95±0.75	20.92±0.32	20.89±0.34
	20 d	24.89±0.33^A^	25.39±1.84^A^	21.15±1.74^B^
	60 d	27.65±0.53^A^	28.55±0.43^A^	24.99±0.54.^B^
Lipase (U/ L)	0 d	19.09±1.53	18.01±0.75	19.09±1.08
	20 d	18.77±1.12^B^	24.31±1.81 ^A^	23.00±1.56^A^
	60 d	21.88±1.93^B^	31.95±3.19^A^	31.52±1.85^A^

^A,B,C^Values with different superscripts in the same row differ significantly (*P<*0.05); I, control group; II and III, groups treated with 2 and 3 g/per head per day of antimicrobial peptides, respectively. (The table are detailed in S1 File)

### 3.3 Rumen microorganisms

#### 3.3.1 Composition of the bacterial communities

In total, we analyzed 820,830 reads for bacteria (a mean of 54,722 reads per sample). The identified bacterial phyla and genera are shown in Tables 5 and 6 (Supplementary Figure A and B in S1 Fig). The results of principal component analysis are detailed in S2 Fig. The main phylum detected in all samples was the Bacteroides, which accounted for on average 42.11% of the bacterial community. The bacterial phyla Proteobacteria and Spirochaetes appeared to decrease in the two AMP-supplemented groups (Table 5), and were lower in the AMP-supplemented goats than in the control group (*P* < 0.05). By contrast, the bacterial phyla Firmicutes, Verrucomicrobia, Tenericutes, Cyanobacteria and Fibrobacteres increased in the AMP-supplemented groups compared with the control (Table 5; *P* < 0.05), but Firmicutes and Verrucomicrobia increased significantly only at 60 days, and in group III only Tenericutes differed significantly from the controls (*P* > 0.05). No differences in the proportion of Bacteroides were observed between the AMP-treated goats and the control group (*P* > 0.05).

**Table 5:** Influence of AMPs on the proportion of different bacterial phyla.

Bacterial phylum		I	II	III
Bacteroidetes	0 d	36.99±1.45	34.35±2.82	36.15±3.77
	20 d	40.87±2.19	41.77±6.26	43.68±3.53
	60 d	47.12±1.10	51.81±4.75	52.77±4.33
Firmicutes	0 d	27.02±4.16	26.75±3.38	28.08±2.50
	20 d	27.19±1.77	30.40±4.44	29.65±3.32
	60 d	18.05±1.07^A^	22.69±0.32^B^	22.70±1.70^B^
Proteobacteria	0 d	19.92±4.13	19.37±2.01	19.69±2.70
	20 d	19.23±2.88^A^	9.31±1.10^B^	7.73±2.46^B^
	60 d	19.99±0.17^A^	8.02±3.28^B^	3.29±0.46^C^
Verrucomicrobia	0 d	4.60±1.67	4.88±1.02	5.06±0.67
	20 d	4.34±0.34	4.85±0.19	4.45±0.40
	60 d	2.69±0.35^A^	4.17±1.51^A^	7.81±2.43^B^
Fibrobacteres	0 d	5.25±0.63	5.76±0.23	5.30±0.62
	20 d	3.93±0.26^A^	5.50±0.46^B^	5.37±0.18^B^
	60 d	2.63±0.40^A^	4.47±0.33^B^	4.36±0.31^B^
Tenericutes	0 d	2.21±0.25	2.16±0.81	2.45±1.48
	20 d	1.83±0.58^A^	2.05±0.54^A^	3.72±0.92^B^
	60 d	2.43±0.44^A^	3.41±0.76^A^^B^	4.56±0.96^B^
Spirochaetes	0 d	0.95±0.15	0.85±0.18	0.69±0.29
	20 d	1.25±0.17^A^	1.15±0.22^A^	0.41±0.08^B^
	60 d	3.00±0.71^A^	1.21±0.41^B^	1.35±0.21^B^
Cyanobacteria	0 d	1.67±0.45	1.53±0.22	1.24±0.39
	20 d	1.13±0.19^A^	2.85±0.48^B^	2.48±0.20^B^
	60 d	0.60±0.11^A^	2.09±0.39^B^	1.45±0.35^C^

^A,B,C^Values with different superscripts in the same row differ significantly (*P<*0.05); I, control group; II and III, groups treated with 2 and 3 g/per head per day of antimicrobial peptides, respectively. (The table are detailed in S1 File)

**Table 6:** Influence of AMPs on the proportion of different bacterial genera.

Bacterial genus		I	II	III
Undefined genera	0 d	39.16±1.83	39.45±3.87	39.66±1.49
	20 d	40.27±2.71	42.38±7.13	39.96±2.76
	60 d	35.57±1.26	36.79±4.04	39.34±1.26
*Prevotella*	0 d	22.2±3.02	21.73±1.29	22.71±2.37
	20 d	25.54±2.66	27.88±0.99	28.71±4.78
	60 d	27.67±2.54	32.48±3.42	32.97±6.85
*[Paraprevotellaceae]CF231*	0 d	7.36±1.53	6.85±1.02	7.71±0.92
	20 d	6.03±1.08	5.25±0.11	5.71±0.81
	60 d	8.79±1.03^A^	4.43±0.69^B^	4.72±0.39^B^
*Butyrivibrio*	0 d	6.51±0.48	7.09±1.43	6.5±0.56
	20 d	6.31±0.86	6.45±0.20	6.52±0.45
	60 d	6.15±0.07	6.60±0.22	6.23±0.17
*Succinivibrioio*	0 d	8.23±1.02	8.07±0.72	7.98±0.34
	20 d	7.56±0.69^A^	1.71±0.39^B^	1.00±0.13^B^
	60 d	3.99±0.52^A^	2.62±0.54^B^	1.33±0.24^C^
*Fibrobacter*	0 d	4.60±0.38	4.90±0.57	4.79±0.45
	20 d	3.60±0.32^A^	5.16±0.16^B^	5.20±0.14^B^
	60 d	2.63±0.40^A^	4.47±0.33^B^	4.36±0.31^B^
*Selenomonas*	0 d	3.39±0.29	3.48±0.34	3.21±0.17
	20 d	2.95±0.16^A^	1.64±0.04^B^	1.75±0.45^B^
	60 d	1.53±0.23^A^	1.09±0.15^B^	0.57±0.16^C^
*Anaerovibrio*	0 d	1.92±0.05	1.99±0.21	2.07±0.14
	20 d	1.48±0.46^A^	0.90±0.12^A^	1.68±0.66^A^
	60 d	1.23±0.27^A^	1.20±0.27^A^	1.65±0.12^A^
*Succiniclasticum*	0 d	1.45±0.21	1.42±0.34	1.57±0.16
	20 d	1.02±0.09^A^	1.27±0.15^B^	1.80±0.09^C^
	60 d	0.04±0.01^A^	1.20±0.40^B^	1.48±0.36^B^
*Treponema*	0 d	0.98±0.13	1.02±0.09	1.15±0.19
	20 d	1.22±0.15^A^	1.00±0.43^A^	0.38±0.11^B^
	60 d	2.95±0.70^A^	1.71±0.14^B^	1.45±0.15^B^

^A,B,C^Values with different superscripts in the same row differ significantly (*P<*0.05); I, control group; II and III, groups treated with 2 and 3 g/per head per day of antimicrobial peptides, respectively. (The table are detailed in S1 File)

At the genus level, *Prevotella* dominated the assignable sequences, on average accounting for 29.21% of the total bacteria. The next most common genera were *Butyrivibrio* (6.38%), [*Paraprevotellaceae*]*CF231* (5.82%), *Fibrobacter* (3.96%), *Succinivibrio* (3.04%) and *Anaerovibrio* (2.63%). The bacterial genera *Fibrobacter* and *Succiniclasticum* were more highly represented in AMP-supplemented goats than in the control group (Table 6; *P* < 0.05). The genera [*Paraprevotellaceae*]*CF231*, *Succinivibrio*, *Selenomonas* and *Treponema* were decreased in the AMP-supplemented goats compared with the control group (Table 6; *P* < 0.05); however, [*Paraprevotellaceae*]*CF231* decreased significantly only at 60 days. No differences in the proportion of *Prevotella*, *Butyrivibrio* or *Anaerovibrio* were evident between AMP-treated goats and the control group (*P* > 0.05). The Simpson and Shannon diversity indexes showed no significant differences between AMP-treated goats and the control group (Tables 7 and 8).

**Table 7:** Diversity estimation based on sequence analysis of the *16S rRNA* gene libraries of the goat rumen^a^.

Item		I	II	III
Reads	0 d	57 339±854	56 724±632	56 872±809
	20 d	55 501±626	54 479±764	59 799±1069
	60 d	57 379±1572	50 764±1037	50 467±970
OTUs	0 d	1 221±101	1 274±68	1 202±144
	20 d	1 211±172	1 251±153	1 192±169
	60d	953±90	1 197±118	1 289±117
Simpson	0 d	0.95±0.049	0.954±0.035	0.949±0.022
	20 d	0.952±0.050	0.973±0.012	0.947±0.25
	60 d	0.950±0.044	0.968±0.040	0.975±0.015
Shannon	0 d	6.561±0.09	6.537±0.12	6.606±0.20
	20 d	6.560±0.14	7.103±0.23	6.573±0.17
	60 d	6.228±0.32	6.835±0.24	7.290±0.25

^a^ Operational taxonomic units (OTUs) were defined with 3% dissimilarity. The diversity indices (Shannon and Simpson) were calculated.

**Table 8:** Diversity estimation based on sequence analysis of the *18S rRNA* gene libraries of the goat rumen^a^.

Item		I	II	III
Reads	0 d	18 916±684	19 392±822	18 536±622
	20 d	18 002±807	22 512±699	18 987±366
	60 d	19 020±513	11 794±71	17 538±633
OTUs	0 d	116±4	138±7	120±4
	20 d	123±3	135±5	130±4
	60d	118±4	135±6	141±5
Simpson	0 d	0.764±0.04	0.77±0.08	0.747±0.01
	20 d	0.766±0.03	0.804±0.06	0.720±0.03
	60 d	0.784±0.02	0.782±0.03	0.769±0.01
Shannon	0 d	2.987±0.12	3.008±0.07	3.019±0.10
	20 d	3.014±0.23	3.117±0.13	2.819±0.26
	60 d	3.081±0.18	3.122±0.14	3.074±0.19

^a^ Operational taxonomic units (OTUs) were defined with 3% dissimilarity. The diversity indices (Shannon and Simpson) were calculated.

#### 3.3.2 Ciliate community structure

A total of 325,008 ciliates reads were retained following filtering to exclude low quality reads, an average of 18,056 reads per rumen sample. Although all animal groups were fed the same diet, a high level of variation was observed between individuals in terms of ciliate community composition at the genus level. The only common characteristic was that *Polyplastron* and *Ophryoscolex* were the dominant ciliates in every sample (Table 9 and Supplementary S3 Fig). The results of principal component analysis are detailed in S4 Fig. The ciliates genus *Ophryoscolex* was increased in AMP-supplemented goats compared with the control group (*P* < 0.05). *Ophryoscolex* replaced *Polyplastron* as the dominant genus in the AMP-supplemented groups. *Polyplastron*, *Entodinium*, *Enoploplastron* and *Isotricha* decreased in the AMP-supplemented goats compared with the control group (Table 9; *P* < 0.05), but in group III animals only *Isotricha* differed significantly from the control (*P* > 0.05). No differences in *Diploplastron* or *Dasytricha* were observed between AMP-treated goats and the control group (*P* > 0.05).

**Table 9:** Influence of diet and AMPs on the proportion of ciliate genera.

Ciliate genus		I	II	III
*Polyplastron*	0 d	40.07±4.31	42.28±3.39	41.23±3.35
	20 d	45.37±0.64^A^	38.06±1.58^B^	33.37±4.71^B^
	60 d	56.78±4.55^A^	43.32±5.21^B^	41.28±1.70^B^
*Diploplastron*	0 d	7.39±1.10	6.82±0.72	6.80±1.12
	20 d	6.17±1.04	5.29±2.41	6.41±0.32
	60 d	3.31±0.54	4.26±0.62	3.36±0.37
*Entodinium*	0 d	4.43±0.92	4.50±0.85	4.12±0.80
	20 d	2.65±0.50^A^	1.77±0.11^B^	0.46±0.16^C^
	60 d	1.38±0.12^A^	0.92±0.32^B^	0.60±0.13^B^
*Ophryoscolex*	0 d	10.86±1.10	10.37±1.78	10.84±0.94
	20 d	14.99±7.23^A^	36.73±8.23^B^	45.07±4.14^B^
	60 d	27.98±3.44^A^	44.07±5.04^B^	52.09±2.13^C^
*Enoploplastron*	0 d	0	0	0
	20 d	0	0	0
	60 d	5.79±1.39^A^	3.13±0.43^B^	0.16±0.14^C^
*Dasytricha*	0 d	0.99±0.16	0.87±0.15	0.79±0.32
	20 d	0.32±0.40	0.29±0.06	0.74±0.32
	60 d	0	0	0.50±0.42
*Isotricha*	0 d	36.09±3.94	35.80±1.93	37.40±2.23
	20 d	29.87±9.69^A^	17.87±5.46^A^^B^	13.95±1.36^B^
	60 d	4.21±0.90^A^	4.18±0.70^A^	2.01±0.46^B^

^A,B,C^Values with different superscripts in the same row differ significantly (*P<*0.05); I, control group; II and III, groups treated with 2 and 3 g/per head per day of antimicrobial peptides, respectively. (The details are in S1 File)

## 4 Discussion

Recently, a large body of research has focused on developing alternatives to antibiotic feed additives. Among these alternatives, AMPs have gained increasing attention because of their broad-spectrum activity, speed of action and low propensity for the development of bacterial resistance[20,32–34]. In general, the development of AMPs into feed addictives has been hampered by their potential for toxic side effects, suboptimal efficacy, and, most notably, the lack of cost-effective production systems.The present study demonstrates the effect of AMPs on different rumen bacteria and ciliates in juvenile goats, which can provide a theoretical basis for the future as alternatives to antibiotic.

In this study, we report that dietary supplementation with AMPs improved the growth of juvenile goats. This was consistent with the finding of Yoon *et al*.[35] who observed an improvement in average daily gain and feed use efficiency in weanling pigs fed diets supplemented with AMP-A3. Similarly, Jin *et al*.[33–34] observed an improvement in average daily gain of weanling pigs fed diets supplemented with AMPs from *Solanum tuberosum*. Moreover, 2 g/head/day of AMPs improved the growth performance more effectively than higher doses (3 g/head/day), although the reason for this remains unclear.

Microbial community composition in ruminants has previously been linked with animal production traits[36]. In the present experiment, we found that Bacteroidetes, Firmicutes and Proteobacteria were the main phyla in all samples. At the genus level, *Prevotella* was the most abundant genus detected, followed by *Butyrivibrio*, [*Paraprevotellaceae*]*CF231*, *Fibrobacter*, *Succinivibrio* and *Anaerovibrio*. Many of these genera include organisms that are important cellulose and hemicellulose-degraders, indicating that the rumen bacterial community may be highly oriented towards fiber degradation. This community structure is similar to the inferred rumen bacterial community structure of sheep[37].

We also found that *Polyplastron* and *Ophryoscolex* were the dominant ciliate genera in all samples. The protozoal community composition was similar to the A type (dominated by *Polyplastron*, *Ostracodinium*, *Dasytricha* and *Entodinium*)[38]. However, other studies have identified *Entodinium* as the most dominant protozoal genus in ruminants[39–42]. This discrepancy between studies may be due to differences in diets. In this study, forage grass was the main fodder supplied, and as a result, the proportions of *Polyplastron* and *Ophryoscolex* were greater than those of *Entodinium*. Dehority and Odenyo[43] reported that the levels of *Entodinium* were considerably higher in animals fed concentrates and intermediate mixed feeds compared with those eating roughage. In addition, high-throughput sequencing might not reflect the true composition of rumen ciliates. Kittelmann *et al*.[44]reported that smaller-celled genera, such as *Entodinium*, *Charonina* and *Diplodinium* tended to be underrepresented, while larger-celled genera, such as *Metadinium*, *Epidinium*, *Eudiplodinium*, *Ostracodinium* and *Polyplastron* tended to be overrepresented using the pyrosequencing approach, indicating that this may not an appropriate methodology in this case.

In goats, growth is accompanied by a decrease in the amount of OTUs, which means a decline in the diversity of rumen bacteria to some degree. On day 60, the number of OTUs in all AMP-treated groups was higher than in the control group, despite a decrease in the abundance of Proteobacteria in two AMP-supplemented groups, which may be explained by the selective effects of AMPs on different bacteria. AMPs provide beneficial effects in host animals by improving their intestinal balance and optimizing the gut microecological conditions that suppress harmful microorganisms, such as *Clostridium* spp. and *coliforms*, and by favoring beneficial microorganisms, such as *Lactobacillus* and *Bifidobacterium*[20,45–47]. A number of recent studies have suggested that dietary supplementation with an AMP, such as lactoferricin and the lactoferrampin fusion peptide, potato protein, antimicrobial peptide P5 or cecropin AD, reduced the total number of aerobes while simultaneously enhancing the total amount of anaerobes and beneficial lactobacilli, thus improving growth performance in weaning pigs[19–20,47–48]. In this study, we report significantly fewer Proteobacteria and significantly more Fibrobacteres in the AMP-supplemented groups. This finding may be explained by Fibrobacteres comprising anaerobic bacteria[49], whereas Proteobacteria comprises aerobic bacteria. Specifically, Proteobacteria includes a number of genera with pathogenic strains[50], and the antibacterial peptide may therefore have inhibited the pathogenic bacteria while enhancing the total number of anaerobes[20]. Dietary supplementation with AMPs increased some bacterial genera and the ciliate genus, whilst also reducing some other bacterial genera and the ciliate genera. Of these, *Fibrobacter*[51–52], *Treponema*[53], *Ophryoscolex*[54], *Enoploplastron*[55] and *Polyplastron*[38] are cellulose-degrading microbes and *Succiniclasticum*[56], *Entodinium* and *Isotricha*[38] are starch-degrading microbes. *Selenomonas* and *Succinivibrio* degrade both starch and cellulose, and *Anaerovibrio*[57] are fat-degrading bacteria. The function of [*Paraprevotellaceae*]*CF231* is unclear, and the levels of *Succiniclasticum*, *Selenomonas*, *Treponema*, *Enoploplastron* and *Entodinium* were low in this study. Therefore, we speculate that, the increase in relative abundance of *Fibrobacter* and *Ophryoscolex* was responsible for the increase in the activities of xylanase and pectinase and the decrease in activity of β-glucosidase in group III, and that the decrease in relative abundance of *Isotricha* was responsible for the decrease in activity of amylase in group III, and the increase in relative abundance of *Anaerovibrio* was the cause of increased lipase activity.

The fermentation products of *Fibrobacter*, *Anaerovibrio*, *Ophryoscolex*, *Polyplastron* and *Isotricha* are acetate, propionate and succinate, those of *Succinivibrio* are succinate, and those of *Butyrivibrio* are acetate and butyrate. Therefore, the increase in relative abundance of *Fibrobacter*, *Anaerovibrio* and *Ophryoscolex* may be the reason for the increase in acetate and propionate. The lack of change in *Butyrivibrio* may be associated with the lack of change in butyrate. Acetate, propionate and butyrate are the main components in VFAs, and account for 95% of the total volatile matter content[58]. Therefore, the cause of the increase in T-VFA may be the same as described above. However, a decline in T-VFA was observed after 20–60 days in both of the AMP-treated groups compared with the control, which may be related to the changing trend of *Fibrobacter*. VFAs, as end-products of fermentation by rumen microorganisms, provide 70%–80% of the calorific requirements of ruminants[59]. The improved growth performance in juvenile goats in the AMP groups might be due to an increase in T-VFAs. This conclusion is consistent with the results reported by Wang *et al*.[60], who showed that a ruminal infusion of soybean small peptide (100, 200, 300 g/day) increased the ammonia, propionate and T-VFA concentration, and improved nutrient digestion and ruminal fermentation in Luxi Yellow cattle. Similarly, Hino *et al*.[61] observed that 12.5–25 mg/L of aibellin enhanced propionate production without significantly affecting the production of T-VFAs, protozoal survival or cellulose digestion *in vitro*. By contrast, Patra *et al*.[62] reported that essential oils (garlic oil, clove oil, eucalyptus oil, oregano oil and peppermint oil) significantly decreased ammonia production, altered the abundance and diversity of archaea, and also exerted adverse effects on ruminal feed digestion and fermentation *in vitro*. The differences in results among studies might be due to variations in the types of additive used, the level of dietary supplementation or the mode of action of the additives.

In summary, this study showed that AMP supplementation maintained the rumen micro-ecological balance, but increased the relative abundance of *Fibrobacter*, *Anaerovibrio* and *Ophryoscolex*, and reduced the relative abundance of [*Paraprevotellaceae*]*CF231*, *Succinivibrio*, *Polyplastron* and *Isotricha*. The supplements also improved the rumen microbiota structure, altered ruminal fermentation, and increased the utilization efficiency of feed, thereby improving the potential growth performance. These results indicated that AMPs can be used as a feed additive in juvenile goats. Aranha *et al*[63]reported that Nisin can inhibit sperm activity in humans, monkeys and mice,and thus we will explore that if longer term use of the AMPs used in the present study can influence the fertility of goats in the future researches. The cytotoxic effects of AMPs on host cells and the detailed mechanism(s) by which AMPs improves the rumen microbiota structure of juvenile goats requires further clarification.

## Supporting information

S1 Fig Relative abundance of bacterial phyla (A) and genera (B) in rumen samples.
(DOC)

S2 Fig The results of principal component analysis(bacterial). (DOC)

S3 Fig Composition of rumen ciliate at genus level. (DOC)

S4 Fig The results of principal component analysis(ciliate). (DOC)

S1 File The parameters of ruminal fermentation,the activity of enzyme and the proportion of bacterial phyla, bacterial genera and ciliate genera. (DOC)
