# Supplementary figures and images for: Effects of antibacterial peptides on rumen fermentation function and rumen microorganisms in goats
Source: PLoS One. 2019 Aug 30;14(8):e0221815. doi: 10.1371/journal.pone.0221815 (PMC6716671; doi:10.1371/journal.pone.0221815)

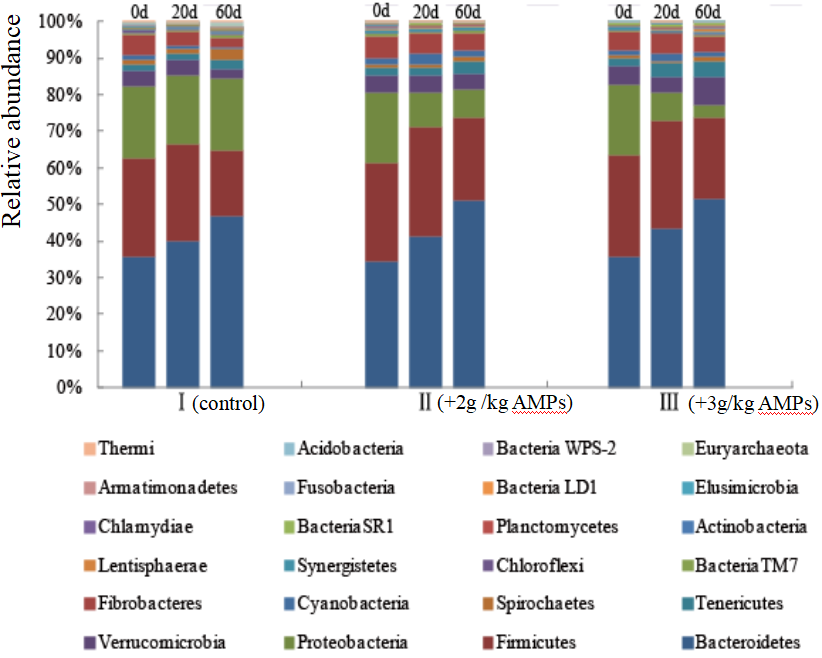

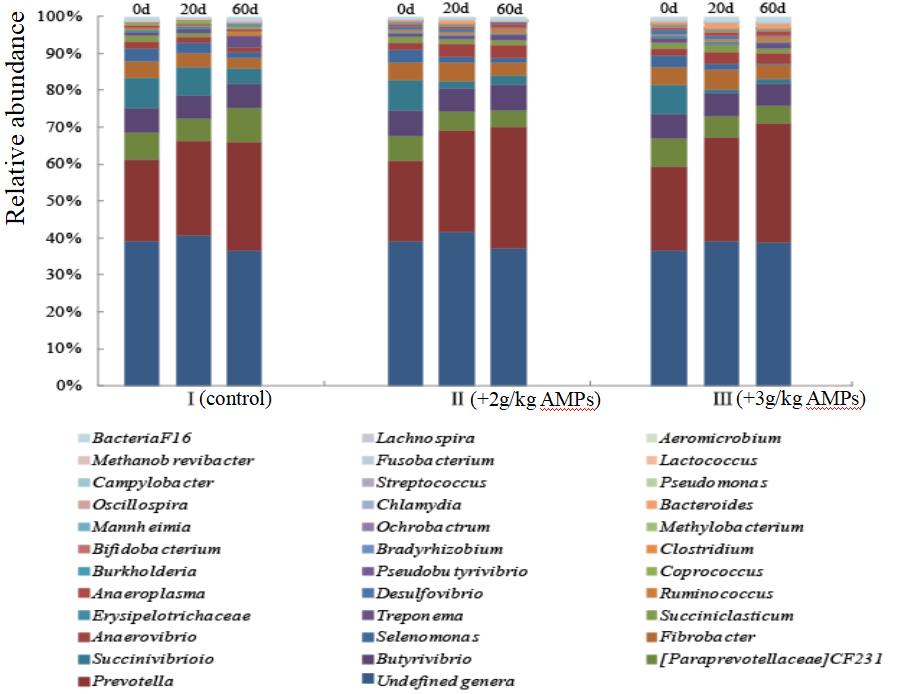


A B

Figure S1. Relative abundance of bacterial phyla (A) and genera (B) in rumen samples

Supplement: S1 Fig — Relative abundance of bacterial phyla (A) and genera (B) in rumen samples. (DOC) [file pone.0221815.s001.doc]

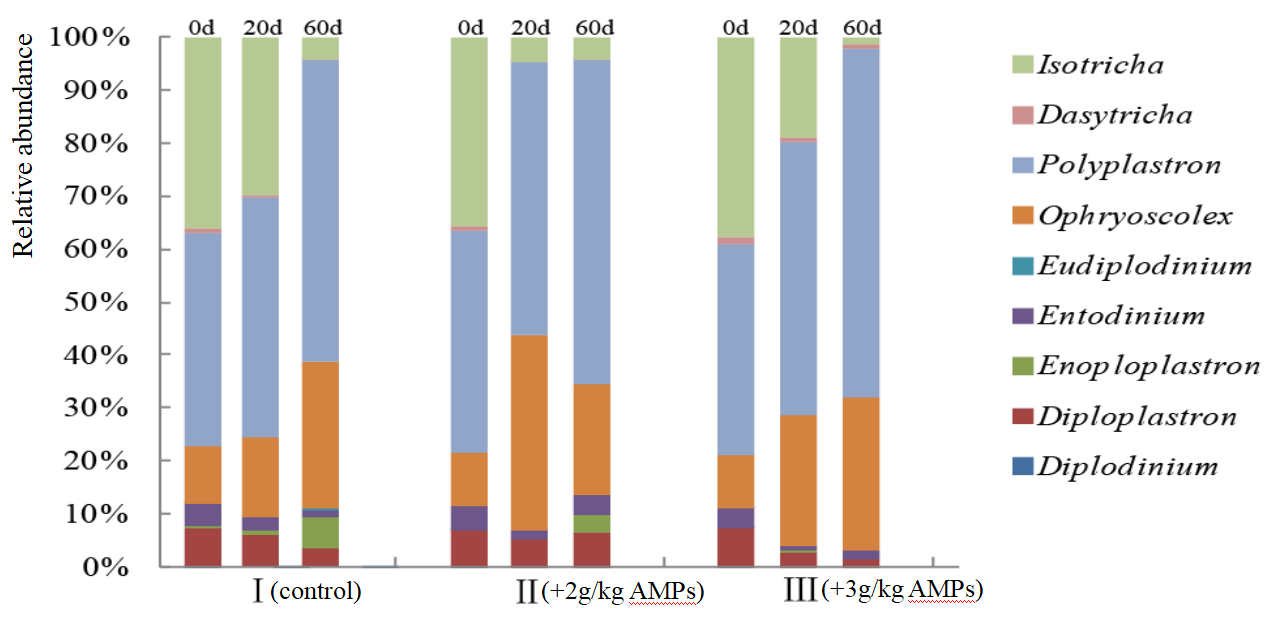


Figure S3. Composition of rumen ciliate at genus level

Supplement: S3 Fig — (DOC) [file pone.0221815.s003.doc]
