# Supplementary material for: Effects of antibacterial peptides on rumen fermentation function and rumen microorganisms in goats
Source: PLoS One. 2019 Aug 30;14(8):e0221815. doi: 10.1371/journal.pone.0221815 (PMC6716671; doi:10.1371/journal.pone.0221815)
Supplement: S2 Fig — (DOC) [file pone.0221815.s002.doc]

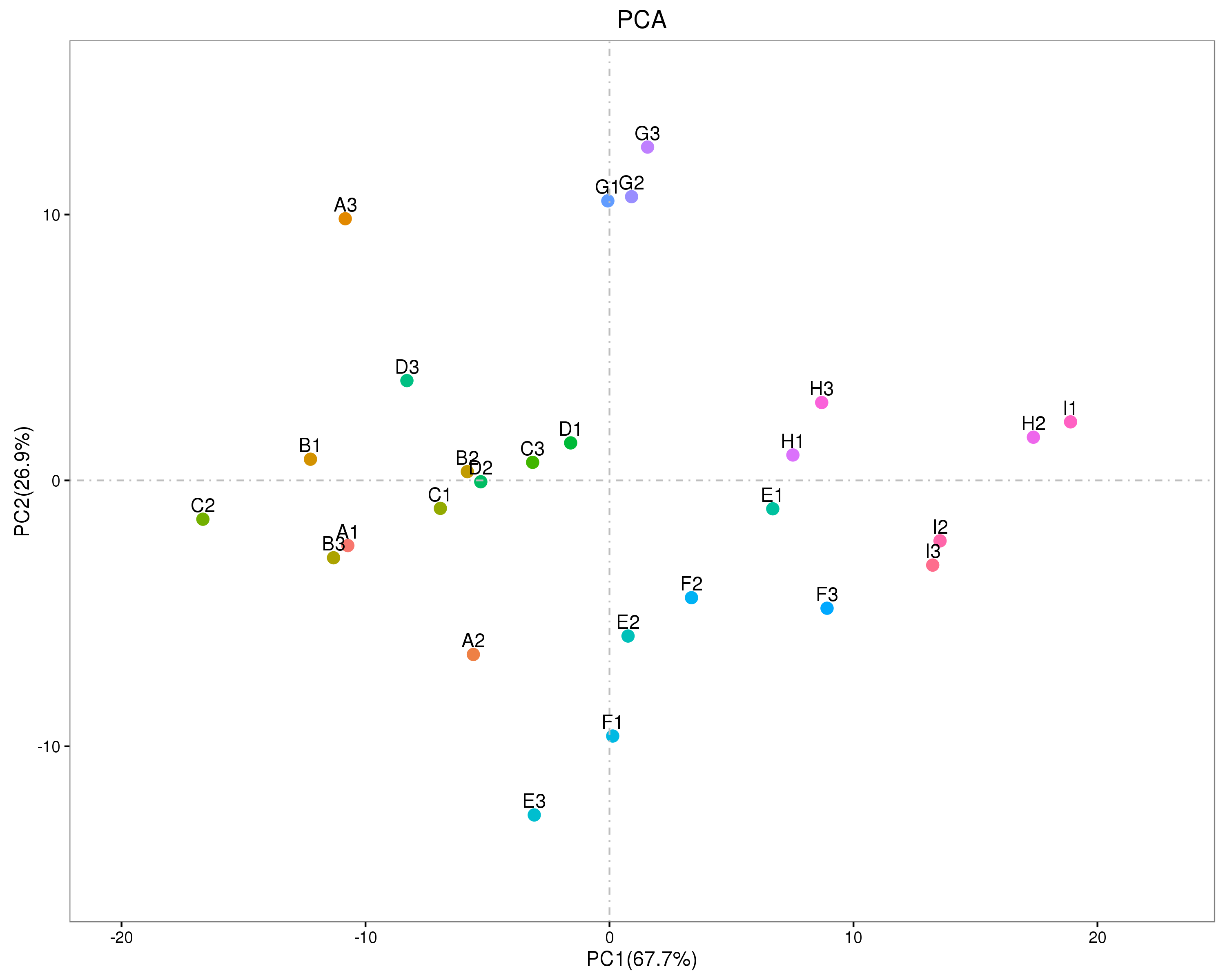


Figure.S2 The results of principal component analysis(bacterial)

As for Figure S2, A、B and C stands for three groups(Ⅰ、Ⅱ、Ⅲ) of 0 days；D、E and F stands for three groups(Ⅰ、Ⅱ、Ⅲ) of 20 days；G、H and I stands for three groups(Ⅰ、Ⅱ、Ⅲ) of 60 days；
