# Supplementary material for: Effects of antibacterial peptides on rumen fermentation function and rumen microorganisms in goats
Source: PLoS One. 2019 Aug 30;14(8):e0221815. doi: 10.1371/journal.pone.0221815 (PMC6716671; doi:10.1371/journal.pone.0221815)
Supplement: S4 Fig — (DOC) [file pone.0221815.s004.doc]

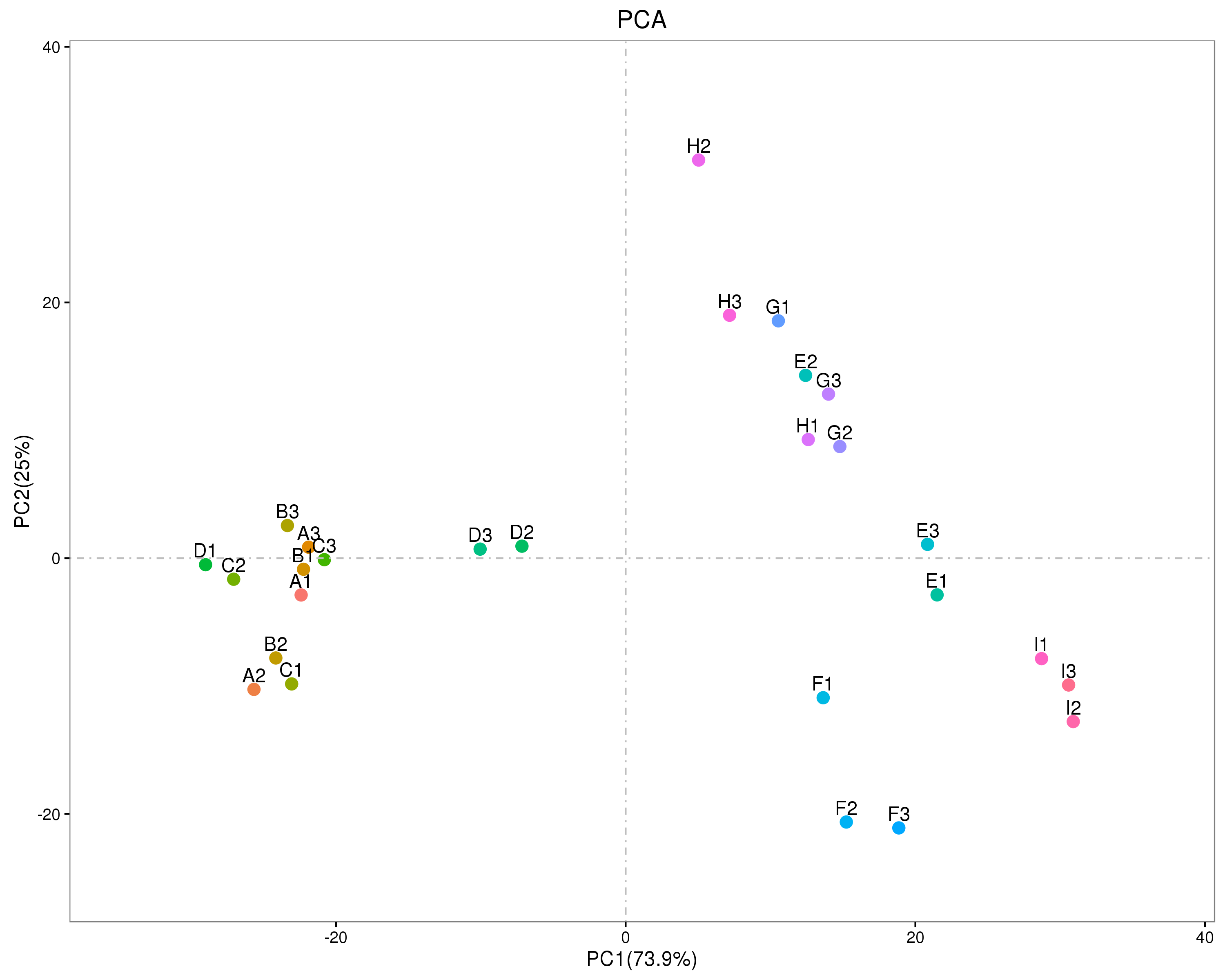


Figure.S4 The results of principal component analysis(ciliate). Sample identifiers are the same as used in Figure S2.
